# Phenolic Alkaloids from *Menispermum dauricum* Rhizome Protect against Brain Ischemia Injury via Regulation of GLT-1, EAAC1 and ROS Generation

**DOI:** 10.3390/molecules17032725

**Published:** 2012-03-06

**Authors:** Bo Zhao, Yang Chen, Xi Sun, Mei Zhou, Jie Ding, Jin-Jin Zhan, Lian-Jun Guo

**Affiliations:** 1 Department of Pharmacology, Tongji Medical College, Huazhong University of Science and Technology, Wuhan 430030, China; Email: cbush2004@163.com (B.Z.); yangchentjyl@163.com (Y.C.); meizhoutjyl@126.com (M.Z.); dingjietjyl@163.com (J.D.); jinjinzhantjyl@126.com (J.-J.Z.); 2 Department of Pharmaceutical Analysis, Drugs Control Centre, Yichang 443002, China; Email: xisunycyj@126.com

**Keywords:** *Menispermum dauricum*, phenolic alkaloids, brain ischemia-reperfusion, glutamate transporter, reactive oxygen species

## Abstract

*Menispermum dauricum* rhizome has been widely used in China to treat various cardiovascular and thrombosis disorders. Some studies have reported that the phenolic alkaloids of *Menispermum dauricum* rhizome (PAM) have protective effects against brain ischemia injury, but the mechanism of this action remains to be clarified. In the present study, we investigated the possible mechanisms of action of PAM on experimental brain ischemia injury. Oxygen and glucose deprivation (OGD) in rat primary cortical cultures and middle cerebral artery occlusion in rats were used to mimic ischemia-reperfusion injury, respectively. The results suggested that PAM protected rat primary cortical cultures against OGD-reoxygenation induced cytotoxicity. PAM decreased extracellular glutamate content and markedly prevented the effects induced by OGD on protein level of GLT-1 and EAAC1 glutamate transporters. In addition, it reduced intracellular ROS generation. *In vivo*, PAM significantly reduced cerebral infarct area and ameliorated neurological functional deficits at different time points. Our findings revealed that the possible mechanism of action of PAM protected against brain ischemia injury involves regulation of GLT-1, EAAC1 and ROS generation.

## 1. Introduction

Cerebrovascular diseases have drawn great public attention recently due to their high death rates and even higher disability rates. Brain ischemia injury is one of the most dangerous diseases in many countries. A restriction of brain blood flow results in stroke and finally leads to neuronal cell death. In China, some 1.5 million people die from stroke each year [1]. Nowadays, as is known that the pathogenesis of brain ischemia-reperfusion (I-R) injury is closely related with excitotoxicity of glutamate and generation of reactive oxygen species (ROS) [2]. 

Glutamate is the most abundant excitatory neurotransmitter in the brain, and a high extracellular level of glutamate release might play an important role in neuronal death [3]. The extracellular glutamate concentration mainly depends on glutamate transporters in astrocyte and neuron, the main influence affected by the activities of two subtypes of glutamate transporter, GLT-1 and EAAC1, which are localized predominantly in astrocytes and neurons, respectively [4,5]. In addition, excessive ROS can lead to oxidative damage of membrane lipid, protein and DNA, causing changes in fluidity and permeability. Thereby, inducing the release of mitochondrial transmembrane proteins to activate apoptotic pathways [6,7]. It is known that the etiopathogenesis of stroke is complicated and the pathogenetic pathways overlap, there are no really effective agents against this disease in clinical practice [8]. Therefore, multi-target drugs from traditional medicine and their respective mechanisms of action are worth investigating.

*Menispermum dauricum* rhizome is a natural product which is widely used in the treatment of cardiovascular and thrombosis disorders in China. Pharmacological research on *Menispermum dauricum* rhizome have shown it to present some biological effects including inhibitory effects on L-type calcium current [9,10], thrombosis and platelet aggregation [11], as well as antiarrhythmic effects [12]. Moreover, *Menispermum dauricum* rhizome protected against brain I-R injury by inhibiting acute inflammation and attenuated the damage of neurons induced by I-R [13,14]. These evidences showed that *Menispermum dauricum* rhizome has protective effects on brain ischemia injury. However, the mechanism of action is still not very clear.

Phenolic alkaloids from *Menispermum dauricum* rhizome (PAM) are among the major pharmacologic constituents of the plant. PAM consists of various fat-soluble bisbenzylisoquinoline alkaloids, mainly dauricine and daurisoline. The content of other examples is minimal [15]. Our previous study has confirmed that PAM has protective effects against heart and brain ischemia injury in rabbits [16]. In this study, we aimed to investigate the protective effect of PAM on rat primary cortical cultures. Meanwhile, we have also explored the mechanism of action of PAM as it correlates with GLT-1, EAAC1 and ROS levels.

## 2. Results and Discussion

### 2.1. Effect of PAM on OGD-Reoxygenation Induced Cell Injury

**Figure 1:**
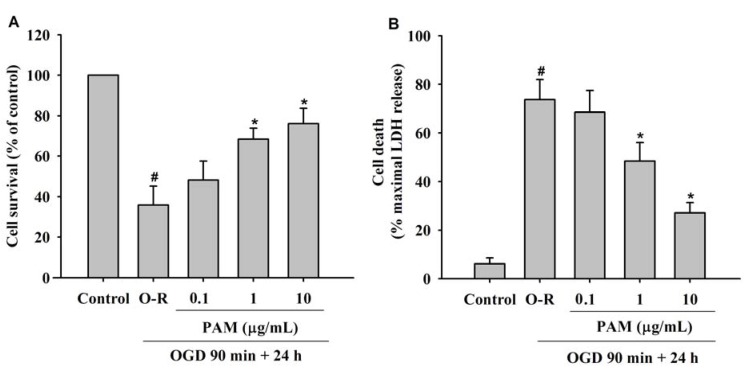
Effects of PAM on cell viability of primary rat cortical cultures. Cell cultures were treated with different concentration of PAM at the start of OGD until the end of reoxygenation. (**A**) Quantitative analysis of cell survival rate was detected by MTT assay; (**B**) Culture supernatants were collected and assayed for LDH with commercial LDH kit. Data are mean ± SD from three independent experiments. The results were compared using ANOVA followed by Fisher’s PLSD test. ^#^ *P* < 0.05 *vs.* control group, * *P* < 0.05 *vs.* O-R group.

Cell survival was determined by measuring methylthiazolyl tetrazolium (MTT) reduction, and cell membrane integrity was determined by lactate dehydrogenase (LDH) leakage. Twenty four h after the cortical cultures were subjected to a 90 min oxygen and glucose deprivation (OGD), 35.84 ± 9.37% of the cells remained viable, as estimated by MTT reduction (Figure 1A and 73.68 ± 8.29% died as assessed by LDH release (Figure 1B). Cell viability increased when PAM (1, 10 μg/mL) were added to the cells at the start of OGD and continued to be present in the cell medium during the reoxygenation period, while LDH efflux was significantly inhibited. These effects were dose-dependent manner.

Astrocytes are known to carry out critical functions such as maintenance of ionic homeostasis and provision of growth factors that potentially may influence the outcome of ischemia injury [17]. On the other hand, GLT-1 protein was undetectable in highly pure cultured astrocytes [18]. According to these evidences, we choose to observe the effects of PAM on primary rat cortical cultures for better simulate the physiological situation. The results revealed that PAM significantly protected primary cortical cultures against OGD-Reoxygenation (O-R) induced injury.

### 2.2. Effects of PAM on OGD-Evoked Increase of Extracellular Glutamate Content

**Figure 2:**
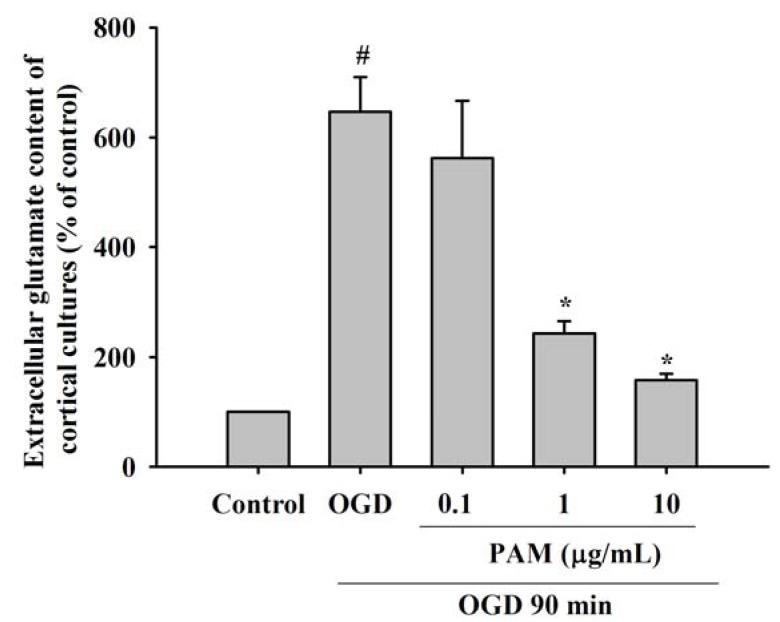
Effects of PAM on extracellular glutamate content of rat primary cortical cultures. At the end of the 90 min OGD exposure, before reoxygenation, extracellular glutamate content was significantly increased compared to those measured in control group. PAM 1, 10 μg/mL significantly inhibited the elevation of glutamate content induced by OGD. Data are mean ± SD from three independent experiments. The results were compared using ANOVA followed by Fisher’s PLSD test. ^#^ *P* < 0.05 *vs.* control group, * *P* < 0.05 *vs.* OGD group.

At the end of the OGD period, extracellular glutamate content in the culture medium was estimated by high-performance liquid chromatography (HPLC) analysis. As shown in Figure 2, at the end of the 90 min OGD exposure, before reoxygenation, extracellular glutamate content of OGD group significantly was increased to 646.91 ± 62.56% in comparison to the control group (*P* < 0.05). Extracellular glutamate content was significantly reduced to 243.10 ± 22.43%, 157.71 ± 11.93% at PAM 1, 10 μg/mL treatment groups comparison with the OGD group (*P* < 0.05). PAM 0.1 μg/mL (562.20 ± 103.29%) did not markedly modify the extracellular glutamate content. 

There is a several-fold increase in extracellular glutamate in brain tissue during *in vivo* and *in vitro* ischemia [19]. Excessive glutamate activated the postsynaptic ionotropic glutamate receptors, which induced intracellular Ca^2+^ overload. Then, it resulted in sustained increase in intracellular Ca^2+^ [20], which was considered a point-of-no-return in triggering cell death [21]. In this study, our data showed that extracellular glutamate content in the culture medium of OGD group was increased after OGD 90 min in cortical cultures. PAM have significant effects against this increase induced by OGD, suggesting that PAM decrease the release of glutamate by presynaptic and/or increase the reuptake in astrocytes.

### 2.3. Effects of PAM on the Level of GLT-1 and EAAC1 after OGD 90 min

**Figure 3:**
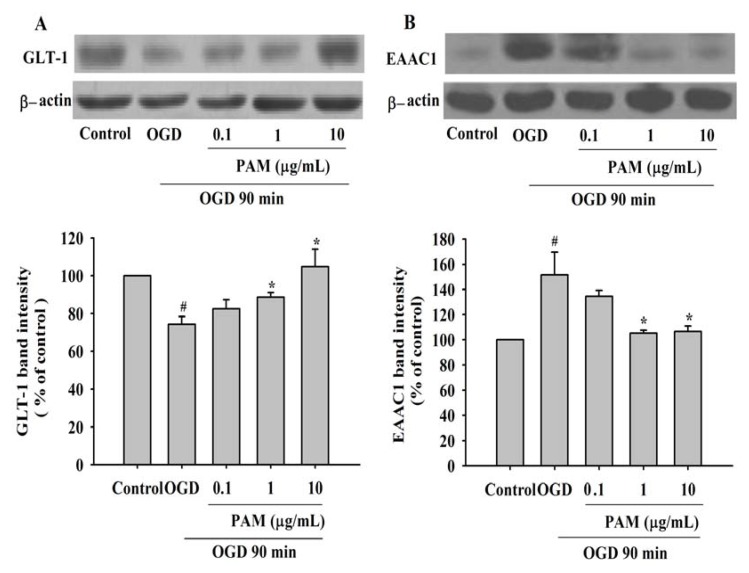
Effects of PAM on GLT-1 and EAAC1 protein expression in rat primary cortical cultures after OGD 90 min. (**A**) Representative Western blot images of GLT-1 and the results of densitometric analysis. (**B**) Representative Western blot images of EAAC1 and the results of densitometric analysis. Actin was used as an internal control. Data are mean ± SD from three independent experiments. The results were compared using ANOVA followed by Fisher’s PLSD test. ^#^ *P* < 0.05 *vs.* control group, * *P* < 0.05 *vs.* OGD group.

Western blot analysis showed that GLT-1 and EAAC1 are expressed in rat cortical cultures (Figure 3). After exposure to OGD 90 min, the protein level of GLT-1 was decreased to 74.32% of control group, whereas the protein level of EAAC1 was increased to 151.61% of control group. Treatment with PAM 1 (10 μg/mL) markedly prevented the effects induced by OGD on GLT-1 and EAAC1. But GLT-1 and EAAC1 protein level were not changed significantly in PAM 0.1 μg/mL treatment group.

Under normal conditions, extracellular glutamate could be transported into intracellular side mainly by GLT-1. However, the function of GLT-1 was impaired when the cell membrane was broken after ischemia, which started with energy depletion-induced intracellular calcium overload and disturbed sodium-potassium exchange. Meanwhile, glutamate release is reversed operated by neuronal EAAC1 in transient cerebral ischemia [22], which means excessive extracellular glutamate in the synaptic cleft, causing excitotoxicity and further impairing homeostasis. Our study showed that the protein levels of GLT-1 were decreased, while the protein levels of EAAC1 were increased after exposure to OGD 90 min in primary cortical cultures. This indicated that PAM was able to exert significant protective effects in primary cortical cultures by regulating GLT-1 and EAAC1. However, it is reported that the protein level of EAAC1 decreased in ipsilateral cortex after transient middle cerebral artery occlusion (MCAO) followed by reperfusion for more than 1 d [23]. This result showed that a delayed down-regulation of EAAC1 induced by transient MCAO was due to decreased number of neurons in the longer reperfusion period. In our study, the cortical cultures were harvested immediately after the end of OGD for Western blot analysis. The early injury induced by transient OGD without reoxygenation is different from the delayed reperfusion injury. This finding indicated that the expression of EAAC1 in early ischemia period may increase, which resulted in large amounts of glutamate released to the synaptic cleft, but EAAC1 still gradually decreased with the extension of injury time.

### 2.4. Effects of PAM on OGD-Reoxygenation Evoked Increase of Intracellular ROS Generation

**Figure 4:**
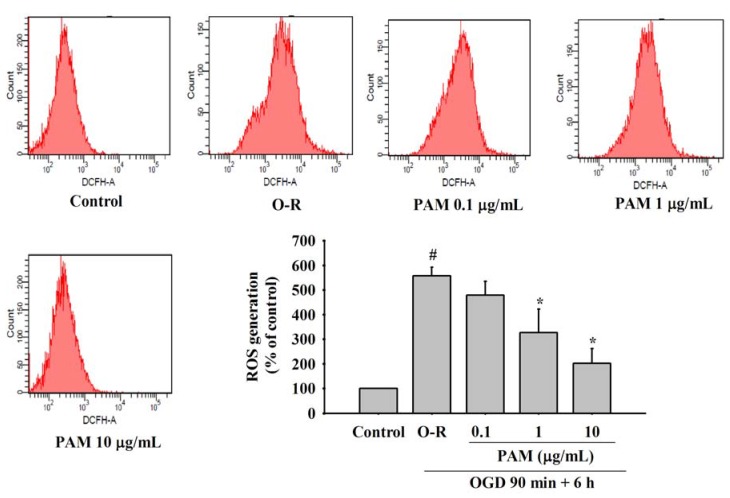
Effects of PAM on OGD-Reoxygenation evoked increase of intracellular ROS generation. ROS alteration was examined by flow cytometry using DCFH-DA after reoxygenation 6 h. Data are mean ± SD from three independent experiments. The results were compared using ANOVA followed by Fisher’s PLSD test. ^#^ *P* < 0.05 *vs.* control group, * *P* < 0.05 *vs.* O-R group.

ROS generation in rat cortical cultures was examined by using flow cytometry. After reoxygenation for 6 h, intracellular ROS generation of O-R group significantly was increased to 556.85 ± 36.09% in comparison to the control group (*P* < 0.05). PAM 0.1 μg/mL treatment group had no significant diminution of intracellular ROS. However, PAM 1, 10 μg/mL treatment groups induced a significant decrease of intracellular ROS to 326.76 ± 96.01%, 202.80 ± 58.44% in comparison to the O-R group (*P* < 0.05) (Figure 4).

ROS plays a major role in biological processes [24], but transient cerebral ischemia significantly increases the generation of ROS such as hydrogen peroxide (H_2_O_2_) and superoxide radical (O_2_^−^) in the cortex during ischemia-reperfusion period [25]. Oxidative stress induced by the formation of ROS exceeds the capacity of antioxidant defense systems [26]. The highly reactive hydroxyl radical is formed from H_2_O_2_ via the Fenton reaction [27]. Once formed, reactive hydroxyl radicals react with many cellular components, which means ROS initiate lipid peroxidation. This process can lead to further ROS generation. In the present study, our data indicated that intracellular ROS generation in primary cortical cultures was markedly increased after the reoxygenation for 6 h. However, PAM can reverse these effects. PAM 0.1 μg/mL treatment group had no significant effect, but improved the effects to a certain extent. Thise evidence revealed that PAM protected against cortical culture injury induced by O-R through scavenging the excessive ROS.

### 2.5. Effects of PAM on Cerebral Infarct Area and Neurological Deficit Score

**Figure 5:**
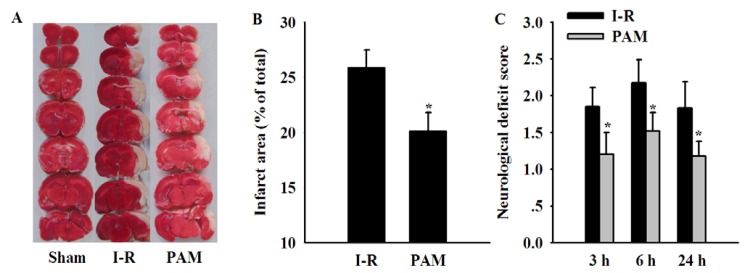
Effects of PAM 10 mg/kg on cerebral infarct area and neurological deficit score. (**A**) Representative images of coronal brain sections stained with TTC; (**B**) Quantitative analysis of infarct area (**C**) Quantitative analysis of neurological deficits score at reperfusion 3 h, 6 h, 24 h, respectively. Data are mean ± SD, n = 8 in each group. The infarct area results were compared using Student’s t-test. Neurological deficit scores were analyzed by Kruskal–Wallis test followed by the Dunn test (multiple comparisons). * *P* < 0.05 *vs.* I-R group.

To assess the protective effects of PAM against brain ischemia injury *in vivo*, a model of transient focal ischemia produced by MCAO was employed. Infarction was assessed by the appearance of a white region after TTC staining at reperfusion for 24 h. The results showed that a marked reduction in the infarct area was observed in PAM 10 mg/kg treatment group (20.1 ± 1.7%) in comparison to the I-R group (25.9 ± 1.6%, *P* < 0.05) at reperfusion 24 h (Figure 5B) and significantly ameliorated neurological deficit scores at reperfusion 3, 6, 24 h, respectively (Figure 5C).

Brain ischemia injury can be induced by a thrombosis, an embolism or systemic hypoperfusion, all of which can cause a restriction of brain blood flow, leading to insufficient oxygen and glucose delivery of brain tissue. The severity of ischemia injury during and following MCAO is determined by the collateral blood supply [28]. Therefore, the first 24 h of reperfusion following MCAO 2 h is critical for tissue survival or demise. Consistent with the *in vitro* experimental results, we demonstrated that PAM significantly improved neurological deficits, and decreased infarct size after MCAO at different reperfusion time points.

## 3. Experimental

### 3.1. Chemicals

2,3,5-Triphenyltetrazolium chloride (TTC), *o*-phthalaldehyde (OPA) were from Sigma (St. Louis, MO, USA). Dulbecco’s modified Eagle’s medium (DMEM) and fetal bovine serum were purchased from Gibco Invitrogen (Carlsbad, CA, USA). Methylthiazolyl tetrazolium (MTT) and mercaptoethanol were obtained from AMRESCO (USA). Cytotoxicity detection kit (Lactate dehydrogenase, LDH) was purchased from Roche (Mannheim, Germany). ROS detection kit was purchased from Beyotime Co. Ltd. (Jiangsu, China). Bicinchoninic acid (BCA) Protein Assay Reagents was purchased from Pierce Biotechnology (Rockford, USA). Anti-GLT-1 rabbit IgG was from Cell Signaling Technology (USA), anti-EAAC1 rabbit IgG was from ABCAM (UK) and anti-β-actin mouse IgG was obtained from Santa Cruz Biotechnologies (USA). Other general agents were purchased from commercial suppliers. PAM is initially dissolved in 0.1 M HCl and then diluted with sterile water to proper concentration (pH 6.5 ± 0.1) as stock solution in cell experiment. The pH value of working solution in the present study does not affect our results. So we did not do a separate vehicle group in the experiments. PAM is initially dissolved in 0.1 M HCl and then diluted with saline to proper concentration as working solution in the *in vivo* experiment.

### 3.2. Preparation of PAM

The dried *Menispermum dauricum* rhizomes were prepared by Dr. Xi-Ping Pan of Department of Botany, Academia Sinica, Yunnan, China. The rhizomes obtained from a local herbal company (Liaoning, China) and identified by Anshan Pharmaceutical Factory (Liaoning, China). The procedure of extracting PAM used in the study was as follows: air-dried and powdered *Menispermum dauricum* rhizome (500 g) was extracted at room temperature with 0.2% H_2_SO_4_ (8,000 mL) and then the extract was alkalinized with ammonia water to pH 8.5. The weight was about 130 g when the separated crude alkaloid was dried. The crude alkaloid was percolated by CHCl_3_ and concentrated. Then the concentrated product was extracted three times with an identical volume of 2% NaOH. The pH value of the alkaline solution was adjusted to 8.5 with 2 mol/L HCl. The ultimate product, a stramineous powder (9.4 g) was obtained when it was dried under vacuum. HPLC analysis revealed that the relative content of each ingredient in PAM was 48.9% daurisoline, 24.7% dauricine, 5.8% guattegaumerine, 2.9% dauricicoline and residue was other fat-soluble alkaloids.

### 3.3. Primary Rat Cortical Cultures and Oxygen-Glucose Deprivation Followed by Reoxygenation

Primary rat cortical cultures were prepared as described in a previous study [29] with slight modifications. In brief, cortex was dissected from the brains of 18-day-old SD rat fetuses and rinsed in ice-cold dissection buffer. Blood vessels, meningeal, striatal, hippocampal tissues, and olfactory bulbs were removed and cortex tissues were mechanically dissociated. Cortex tissues were treated in supplemented Hank’s balanced salt solution for 15 min at 37 °C. Trypsinized cells suspensions were centrifuged at 968 ×g for 10 min and the pellets were resuspended in DMEM supplemented with 10% fetal bovine serum, 100 U/mL penicillin and 100 μg/mL streptomycin. Cells were seeded at a density of 1.0 × 10^5^ cells/cm^2^ on 96-well plate or 50 mL flask previously coated with poly-D-lysine and were incubated in a humidified incubator with 5% CO_2_ at 37 °C. Half of the culture medium was changed every 2 days. Experiments were performed on mature cultures, in a serum-free medium, at 13 days *in vitro*.

Cortical cultures were exposed to a transient OGD as described previously [29]. Cell cultures submitted to OGD were incubated in glucose-free balanced salt solution containing (mmol/L): NaCl 116, KCl 5.4, MgSO_4_ 0.8, NaH_2_PO_4_ 1.0, CaCl_2_ 1.8, and NaHCO_3_ 26 and bubbled with a gas mix (95% N_2_, 5% CO_2_) for 30 min to remove residual oxygen. Then, the cells were placed in an anaerobic chamber filled with 95% N_2_ and 5% CO_2_ at 37 °C for 90 min. Control cultures, not subjected to OGD, were incubated with balanced salt solution containing 20 mM glucose and maintained in an incubator in air with 5% CO_2_ at 37 °C. OGD was terminated by returning back to the oxygenated DMEM supplemented with 20 mM glucose under normoxic conditions for 24 h. PAM (0.1, 1, 10 μg/mL) were added to the culture medium at the start of OGD until the end of reoxygenation.

### 3.4. MTT Reduction Test and LDH Assay

Cell survival was determined by the MTT assay as described previously [30]. At the end of experiment, the cells in 96-well plates were incubated with MTT (0.5 g/L, 200 μL per well) at 37 °C for 4 h. Then the medium was carefully discarded and 120 μL dimethyl sulfoxide (DMSO) per well was added to dissolve the blue formazan product. The values of absorbance at 570 nm with background subtraction at 630 nm were measured using an ELISA reader (TECAN A-5082, Megllan, Austria). The results of the absorbance of the test wells were expressed as percent of the control group.

At the end of experiment, the medium was collected and centrifuged at 4840 × g for 10 min at 4 °C. The supernatant was collected for the assay and the protein level was measured by BCA method. LDH leakage in the supernatant was measured according to the direction of cytotoxicity detection kit. The value of absorbance was measured at 492 nm using an ELISA reader. The results of the absorbance of the test wells were expressed as percent of the maximal LDH release.

### 3.5. Glutamate Assay in the Culture Medium

According to the methods described previously [31], extracellular glutamate content in the culture medium was determined by HPLC with fluorescence detection using a spectrophotometer (excitation wavelength 330 nm, emission wavelength 420 nm, WATERS 2475 multi fluorescence detection, Milford, MA, USA) after automatic precolumn derivatization with OPA at the end of OGD period. The derivatization of samples and standards was carried out in an autosampler (WATERS). The sample injection volume is 10 μL. Derivatized samples were separated on a Sunfire C-18 column (5 μm, 4.6 mm × 150 mm, WATERS) with the mobile phase consisted of Buffer A: 0.075 M PBS (pH 6.8) and Buffer B: pure methanol, flow rate 1 mL/min and the elution time was 15 min (A:B = 6:4, v/v). Data were collected and analyzed using the Empower software. External standard curve was used to quantify the glutamate content according to peak area. The content of glutamate was calculated according to the standard curve, expressed as percent of the control group.

### 3.6. Western Blot Analysis of GLT-1 and EAAC1

At the end of the OGD period, the protein levels of GLT-1 and EAAC1 in the cortical cultures were analyzed by Western blotting. The cultures were washed with ice-cold PBS and harvested in lysis buffer containing 50 mM Tris, 150 mM NaCl, 0.1% SDS, 1% Nonidet P-40, 0.5% sodium deoxycholate (pH 8.0), and a protease inhibitor cocktail. The cellular debris was removed by centrifugation at 9,680 ×g for 15 min at 4 °C and the total protein content was measured by BCA method. The proteins were size-separated in 10% sodium dodecyl sulfate-polyacrylamide gel electrophoresis and transferred to polyvinylidene difluoride membranes (Hybond-P, GE Healthcare, Little Chalfont, Buckinghamshire, England). The membrane was then incubated at 4 °C overnight in tris-(hydroxymethyl)-aminomethane buffered saline (TBS) containing 5% milk and different primary rabbit antibody against GLT-1 (1:1000 dilution) and EAAC1 (1:100 dilution), respectively. After washing with TBS, the membranes were incubated with horseradish peroxidase-conjugated secondary antibodies (1:10000) at room temperature for 1 h and visualized with an enhanced chemiluminescence system (ECL kit, Pierce Biotechnology, Rockford, IL, USA). The expression in each sample was analyzed with Image J software and quantified after contrasting with β-actin. The bands intensity was expressed as percent of the value of the control group.

### 3.7. Intracellular ROS Measurement

The production of intracellular ROS was measured by dichlorofluorescein diacetate (DCFH-DA) probe according to manufacturer's instructions. The DCFH-DA probe passively diffuses into cells in which it is hydrolyzed by intracellular esterase to DCFH. Then the DCFH was converted by intracellular ROS to fluorescent compound DCF. After reoxygenation 6 h, the cortical cultures were harvested and incubated with 10 μM DCFH-DA for 30 min at 37 °C. Then the fluorescence intensity of DCF was measured at 488 nm for excitation and 525 nm for emission using flow cytometry (BD Biosciences, San Jose, CA, USA). The ROS generation was expressed as percent of the value of the control group.

### 3.8. Animals

Adult male Sprague-Dawley rats weighing 220–250 g were from the Experimental Animal Center in the Tongji Medical College of Huazhong University of Science and Technology (Wuhan, China). The animals were maintained on a 12 h light/dark cycle and had no restriction to eating food or drinking water. All experiments were performed in accordance with the Guidelines of the Care and Use of Laboratory Animals of Tongji Medical College, Huazhong University of Science and Technology. Efforts were made to minimize animal suffering.

### 3.9. Cerebral Ischemia-Reperfusion Model in Rat and Drug Administration

The middle cerebral artery was occluded with a 4-0 silicone-coated nylon suture (18 mm) by surgical operation [32]. In brief, the rats were anesthetized with Chloral hydrate (300 mg/kg, i.p.). Body temperature was maintained at 37 ± 0.5 °C with a heating lamp throughout the anesthetic period. The right common carotid artery (CCA), external carotid artery (ECA) and internal carotid artery (ICA) were isolated. The nylon suture (diameter 0.23 mm) with its tip rounded by heating near a flame was introduced into the ECA lumen and advanced into the ICA until it blocked the origin of the middle cerebral artery (MCA). Reperfusion was induced after 2 h MCAO by suture withdrawal for 24 h. Sham-operated animals were subjected to the same surgical procedure, but the suture was not advanced beyond the internal carotid bifurcation.

The rats were randomly divided into three groups: sham-operated group, I-R group, I-R with PAM 10 mg/kg treatment group (each group n = 8). Due to a study of dose-effect relationship of PAM [33], we choose PAM 5, 10, 20 mg/kg to perform study. The aim of our experiment is to explore the mechanism *in vitro*, and then verifies the validity of PAM *in vivo*. So we only choose the middle dose to reduce the number of animals. PAM was injected intraperitoneally one time at the start of reperfusion and another one at the 22 h after reperfusion. Sham-operated group and I-R group give the corresponding volume of saline at the same time.

### 3.10. Neurological Deficit Evaluation and Infarct Size Measurement

Neurological deficit scores were evaluated after reperfusion 3 h, 6 h, 24 h as described previously [32] on a five-point scale (grade 0: Showing no observable deficit; grade1: A failure to fully extend left forepaw; grade 2: Circling to the left; grade 3: Falling to the left; grade 4: No walking spontaneously and having a depressed level of consciousness). The evaluation was performed by an observer who was blind to the group.

Cerebral infarct size was determined by TTC staining. At the end of reperfusion, the rats were sacrificed under anesthesia, and then brains were removed immediately and sectioned into consecutive 2-mm-thick coronal slices using a vibratome (Campden Instruments, Lafayette, LA, USA). Slices were immediately immersed in 2% TTC medium at 37 °C for 15 min. Stained slices were washed in phosphate buffer saline (PBS) for 5 min and fixed in 4% paraformaldehyde solution for 24 h. After staining and fixation, color images of these slices were captured using a video camera (Olympus, Tokyo, Japan) and analyzed for the infarct area using the Image-Pro plus 5.0 analysis software. The percentage of infarcted volume was calculated as described [34]: [(VC-VL)/VC] × 100%, which VC is the volume of control hemisphere and VL is the volume of non-infarcted tissue in the lesioned hemisphere.

### 3.11. Statistical Analysis

All results are expressed as mean ± S.D. The results were compared using ANOVA followed by a Student’s t-test, Fisher’s PLSD test (SPSS 13.0). Neurological deficit scores were analyzed by Kruskal–Wallis test followed by the Dunn test (multiple comparisons). Differences were considered significant for *P* < 0.05.

## 4. Conclusions

In conclusion, the data suggested that PAM has protective effects on brain ischemia-reperfusion injury *in vitro* through regulation of glutamate transporters expression and intracellular ROS generation. *In vivo*, PAM 10 mg/kg reduced the infarct volume and ameliorated neurological deficit scores. Whether PAM is acting in the same mechanism as in the *in vitro* model still needs further experimental conformation. These findings suggested that PAM might serve as a promising agent against brain ischemia injury.
